# Leaning left: how turn bias and habitat complexity shape spatial cognition in a coral-reef fish

**DOI:** 10.1007/s10071-026-02116-7

**Published:** 2026-10-11

**Authors:** Helen von Drenkmann, Annabell Klinke, Culum Brown

**Affiliations:** 1 School of Natural Sciences, Macquarie University, Sydney, NSW Australia; 2 Humboldt-Universität zu Berlin, Institut für Biologie, Berlin, Germany

**Keywords:** Spatial cognition, Habitat complexity, Navigation strategies, Egocentric and allocentric cues, Lateralisation

## Abstract

In coral reef ecosystems, anthropogenic change is rapidly altering habitat structure, but the consequences for fish cognition remain poorly understood. We tested whether structural habitat complexity influences spatial learning and strategy use in the humbug damselfish (*Dascyllus aruanus*). Juveniles were reared in aquaria containing either high- or low-complexity coral structures and trained in a T-maze task, requiring the use of egocentric (turn-based) and/or allocentric (landmark-based) strategies to locate a reward. Performance was assessed using the number of trials to reach a learning criterion and latency to make correct decisions. Probe trials with conflicting cues assessed navigation strategies. Turn direction was the strongest predictor of performance. Fish trained to turn left reached criterion in fewer trials and consistently relied on egocentric strategies, indicative of a lateralisation bias. In contrast, right-turning fish increasingly relied on landmark cues during probe trials and made faster decisions when doing so, suggesting that allocentric strategies reduced the cost of acting against intrinsic turn biases. Contrary to our expectation, environmental complexity did not affect the number of trials required to reach criterion. However, high-complexity fish located the reward faster during early training and showed longer decision latencies during probe trials, suggesting greater sensitivity to landmark information when cues conflicted. Our results suggest that spatial task performance may be shaped more by turn biases and strategy use than by environmental complexity alone, highlighting the importance of considering intrinsic behavioural constraints when interpreting spatial cognition.

**Supplementary Information:**

The online version contains supplementary material available at https://doi.org/10.1007/s10071-026-02116-7.

## Introduction

The Anthropocene is characterised by rapid environmental change, including global warming and habitat degradation (Hughes et al. 2017; He and Silliman 2019), which alter habitat structure and the spatial cues animals rely on for navigation. Coral reef ecosystems provide a particularly relevant case, as structural complexity is closely linked to biodiversity by providing shelter, spawning, and feeding sites (Graham and Nash 2013; Spalding and Brown 2015). Climate-driven coral mortality and erosion, in combination with rising storm intensity, reduce reef three-dimensional structure in a process known as reef flattening (Alvarez-Filip et al. 2009; Spalding and Brown 2015; Magel et al. 2019). While large-scale ecological consequences of habitat degradation are well documented (Pratchett et al. 2008; Habibullah et al. 2022), far less is known about its effects on cognition and behaviour; which may precede or drive broader ecological shifts.

The ecological cognition hypothesis (Real 1993) proposes that cognitive abilities and neural traits are shaped by the specific demands of an organism’s habitat. Although the hypothesis has an evolutionary basis, environmental conditions can also shape the development and expression of neural and cognitive traits through cognitive plasticity (Fong et al. 2019; Ebbesson and Braithwaite 2012; Klinke and Brown 2026). Exposure to different levels of environmental complexity may therefore result in differences in cognitive performance of a population within a single generation. The effects of habitat complexity on learning has been studied extensively, with reviews by Shumway (2008) and, more recently, Klinke and Brown (2026).

Teleost fishes are particularly useful models for studying the relationship between environmental complexity and cognition because their brains remain responsive to environmental conditions throughout life and exhibit substantial neuroplasticity (Shumway 2008; Zupanc and Sîrbulescu 2011; Ebbesson and Braithwaite 2012). A number of studies provide evidence that environmental complexity can influence neural development and cognitive performance in fishes. For example, guppies raised in enriched tanks develop relatively larger brains and process information more quickly than those raised in barren tanks (Iffert and Stein 2024), climbing perch from structurally complex stream habitats learn spatial routes faster than conspecifics from ponds (Sheenaja and Thomas 2011), and amphibious killifish with terrestrial experience outperform aquatic-only individuals in spatial tasks (Rossi and Wright 2021).

Spatial cognition might be particularly sensitive to changes in habitat complexity, since environmental features can impact navigation and spatial learning. For most species, biologically relevant locations and reference points in the environment are subject to varying degrees of change (Odling-Smee and Braithwaite 2003b). Efficient navigation and memory for locations are therefore fundamental to many life-history processes such as foraging, reproduction, competition and predator avoidance (Brown 2003; Odling-Smee and Braithwaite 2003b; Shettleworth 2010). Animals use different spatial navigation strategies to achieve this (López et al. 1999). In egocentric localisation, the animal encodes spatial information with respect to itself, using an internal sense of the direction and distance of a target from its current position. In contrast, allocentric localisation relies on stable external frames of reference, such as landmarks in the environment (Shettleworth 2010). In laboratory settings, spatial cognition is often investigated using maze experiments, in which individuals must locate a reward repeatedly across trials. The T-maze paradigm is among the most widely used assays of spatial learning and has been successfully employed across a broad range of taxa to examine learning performance, and navigation strategies. Subjects may solve this task either by learning a sequence of body turns (egocentric) and/or by using landmarks (allocentric). Evidence from rodents suggests that different spatial cues are processed hierarchically, with egocentric sensory and motor information providing key inputs to form an allocentric representation of the environment (Wang et al. 2020). This implies that egocentric mechanisms may form a subsystem of allocentric navigation (Sibeaux et al. 2025).

Environmental context can shape which spatial strategies animals predominantly rely on (Rodríguez et al. 2021). Previous studies suggest that in barren or dynamic environments, where physical features can change unpredictably, fish mostly use egocentric strategies, while individuals from landmark-rich and stable environments rely on both turn-direction and landmark cues (Girvan and Braithwaite 1998; Odling-Smee and Braithwaite 2003a; White and Brown 2015). On intact coral reefs, the high structural complexity offers abundant, stable landmarks that can be used in allocentric navigation. Coral reef fishes have indeed been shown to use landmarks to navigate within their home ranges or during migration (Reese 1989; Mazeroll and Montgomery 1998). However, on degrading reefs, formerly reliable landmarks may become sparse or unstable; for example, coral rubble that is displaced by wave action, currents, or tropical storms (Le Quesne et al. 2025). Thus, the decrease in habitat complexity caused by reef flattening could not only affect brain development and cognition of coral reef fishes, but potentially also shift the relative benefits of egocentric and allocentric navigation strategies.

Environmental factors can also influence brain function; for example, lateralisation in rainbowfish and zebrafish (Bibost et al. 2012; Gjinaj et al. 2026). Cerebral lateralisation is the tendency for one brain hemisphere to be structurally different from the other and/or perform a different set of functions (Bisazza and Brown 2011). The most familiar example of lateralisation is human handedness, but it is a widespread phenomenon across vertebrates and may be manifested as consistent turn biases in fishes (Brown and Braithwaite 2005). It is thought that the advantage of lateralisation lies in the splitting of tasks between the two brain hemispheres, which allows for parallel processing and reduced interference between tasks (Rogers et al. 2004; Bibost and Brown 2014). However, there can be costs, for example when tasks require hemispheric cooperation (Dadda et al. 2009), or in situations where individuals must act against their natural lateralised bias (Brown and Braithwaite 2005). Consequently, lateralisation may be a source of individual differences in spatial cognition and motor biases could be relevant in tasks that require using egocentric strategies.

The humbug damselfish, *Dascyllus aruanus* (L. 1758) is common throughout Indo-Pacific coral reefs. Individuals form social groups with limited home ranges centred around a single coral colony (Sale 1971). Humbugs exhibit strict dominance hierarchies (Fricke 1977), and social groups form stable units that can persist for months (Forrester 1991). Group size is closely linked to the size and quality of available shelter in the form of branching corals such as *Acropora* and *Pocillopora* (Forrester 1991; Pratchett et al. 2012). Despite their affinity for live coral, humbugs are found on both intact coral and coral rubble particularly after catastrophic events such as cyclones and bleaching. Thus, humbugs are likely experiencing big shifts in habitat complexity over short periods of time. Given their strong site fidelity and reliance on coral structures as habitat, humbugs are ideal models for assessing how reef degradation may affect cognition.

This study aimed to investigate how ecologically realistic variation in habitat structure impacts spatial learning in humbug damselfish. Reef degradation rarely results in the complete loss of physical structure. Instead, intact branching corals are often replaced by rubble fields and fragmented coral remains (Alvarez-Filip et al. 2009; Magel et al. 2019). Accordingly, fish were reared in aquaria containing either branching coral (high complexity) or rubble (low complexity) structures. They were then tested in a spatial learning task requiring them to learn a reward location across repeated trials in a maze. Fish were trained to obtain the reward by consistently turning either left or right at the maze choice point, while landmarks provided an alternative allocentric cue. We used two behavioural metrics to examine spatial learning competence: learning speed measured as trials to learning criterion, and latency to make a correct decision during each trial. Based on the ecological cognition hypothesis, we predicted that fish reared in more complex habitats would reach the learning criterion in fewer trials and make more correct decisions with shorter latencies than fish from low-complexity environments. Because complex habitats contain stable landmarks and impose higher navigational demands, they should favour the use of allocentric spatial strategies, whereas simpler environments may favour egocentric strategies. We therefore predicted that high-complexity fish would rely more strongly on landmark cues. Fish were randomly assigned to learn either a right or left turn. Because egocentric strategies rely on repeated body turns and fishes can show turn biases, individual variation in lateralisation may be an important determinant of spatial learning performance. We therefore also assessed whether the direction fish were trained to turn affected learning performance and navigation strategy.

## Methods

### Study animals and environmental treatment

**Fig. 1:**
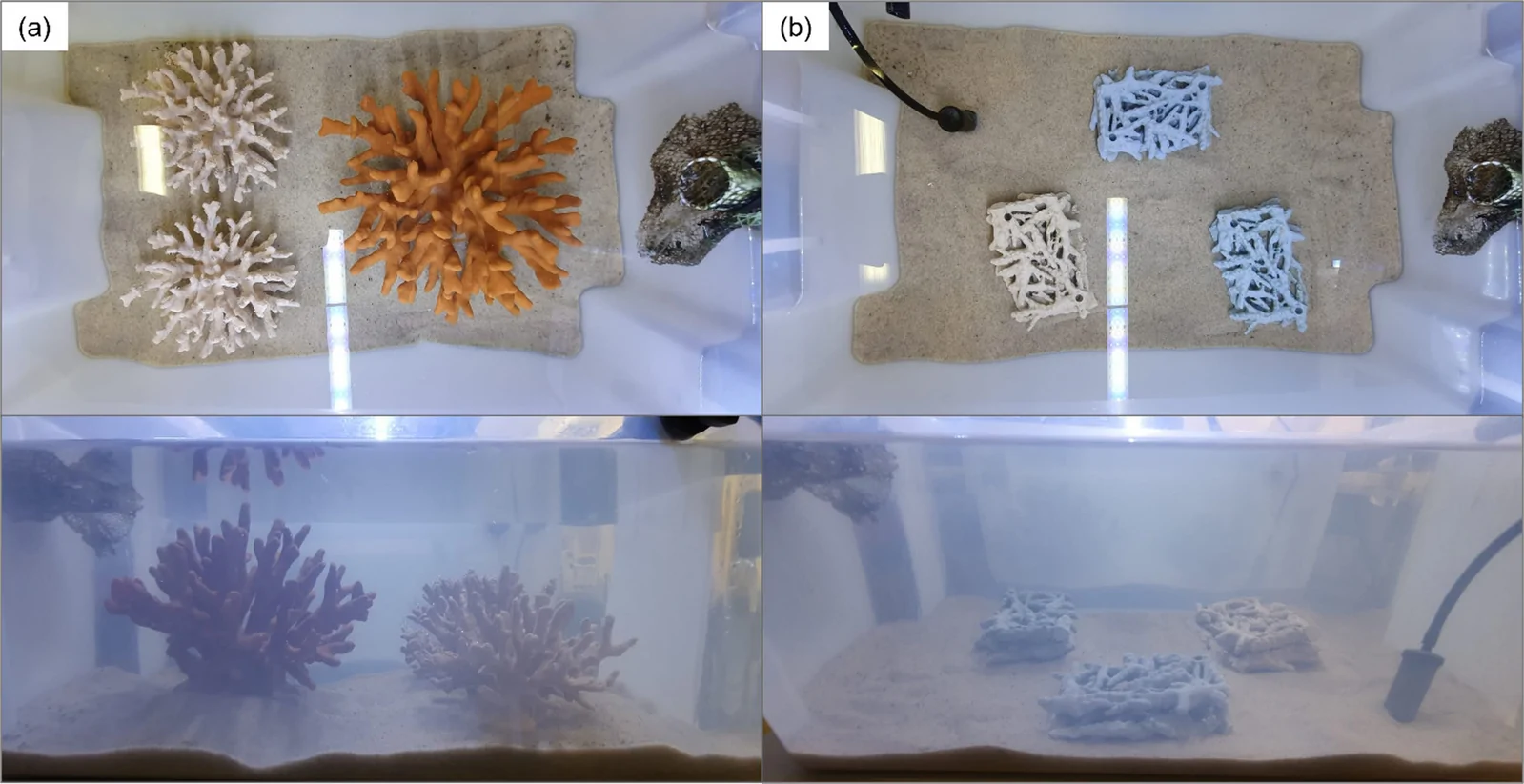
Overhead and side views of 3D-printed coral enrichment used in the environmental treatments. (**a**) High-complexity (HC) treatment. (**b**) Low-complexity (LC) treatment

All experiments were conducted at the seawater facility of the Behaviour, Ecology and Evolution of Fishes Laboratory, Macquarie University (Sydney, NSW, Australia). Juvenile *D. aruanus* were obtained from Elford Reef via Cairns Marine Pty Ltd (QLD, Australia) in May 2024 and housed in groups of 3–5 individuals in eight flow-through seawater aquaria (55 × 37 × 27 cm). Seawater was delivered from Sydney Harbour and mechanically and biologically filtered, as well as UV sterilized. Water temperature was maintained at 27 °C and salinity at 33.1 ppt. Tanks were illuminated with full-spectrum UV lights on a 12:12 h light: dark cycle to simulate tropical conditions (Aqua One StripGlo Sunlight LED Reflector 21 W 90 cm). Each tank was equipped with an air stone, natural sand substrate and three pieces of 3D-printed coral enrichment made from polylactic acid (PLA). Four tanks contained enrichment resembling intact, branching corals (high-complexity treatment, HC), and the remaining four tanks contained coral rubble-shaped structures (low-complexity treatment, LC) (Fig. 1). The HC structures were based on the coral-like models described by Oren et al. (2023), with each tank containing two structures of the original size (approximately 160 × 160 × 113 mm) and one structure 1.5 times larger. The LC structures were based on the artificial coral rubble models described by Wolfe and Mumby (2020), with each structure consisting of two stacked layers, each approximately 140 × 90 × 20 mm. Fish used in the experiment ranged from 3.0 to 5.4 cm standard length. The HC structures provided sufficient space for fish to enter and shelter within them, whereas the smaller openings among the LC pieces provided only limited shelter for smaller individuals.

Fish were raised under these differential environmental treatments for approximately one year prior to the spatial learning task, providing prolonged exposure before testing. It is worth noting that due to observations of aggressive behaviour, HC tanks housed no more than three fish per tank (*n* = 12; 4 tanks x 3 fish), while LC tanks contained 4–5 fish per tank (*n* = 17; 3 tanks x 4 fish, 1 tank x 5 fish). The greater aggression observed in HC tanks may have been related to greater availability and quality of shelter, which could provide resources that individuals are motivated to defend; however, aggression was not quantified in the present study and this potential explanation was not directly tested. The resulting differences in housing density were unavoidable given logistical and financial constraints that prevented the use of additional tanks, but should be considered a possible caveat when interpreting treatment effects.

Fish were fed with a combination of commercial dry pellets (New Life Spectrum^®^ Marine Fish, USA) offered twice daily and frozen adult brine shrimp (*Artemia* sp.) offered three times per week in the afternoon. All fish were tagged using visible implant fluorescent elastomer tags (VIE; Northwest Marine Technology, Inc., USA) to enable individual identification. Standard body length was first measured in October 2024 and again in May 2025. Individuals were assigned to one of three social ranks (dominant, intermediate, or subordinate), primarily based on relative body size, as larger individuals of this species are typically occupy dominant ranks. During routine daily feeding, we observed aggressive encounters including chasing, biting, and priority access to food, which were consistent with the assigned social hierarchy. These observed interactions were merely qualitative and not systematically quantified as a measure of aggression. Information on individual fish, including social rank, can be found in the Supplementary Materials.

### Spatial learning task

To ensure balanced representation across treatments and social ranks, 20 subjects were chosen for the spatial learning task, including 10 HC and 10 LC fish (4 dominant, 3 intermediate, 3 subordinate individuals from each treatment group).

The apparatus and procedures were based on those used by Odling-Smee and Braithwaite ([Bibr CR41][Bibr CR41]) and White and Brown (2015). An X-maze constructed from 3-mm polyvinyl chloride (PVC) was submerged to a depth of 10 cm in seawater within a circular pool (80 cm diameter, 30 cm height). The seawater came from the same reservoir used for the home tanks and was aerated with bubble stones and maintained at 27 °C using heating rods. Grooves along the maze walls allowed a removable opaque PVC screen to be inserted, converting the X-maze into a T-maze with 3 arms. Similar partitions were also positioned in the maze in the middle of each arm to create reward compartments. A doorway was located mid-way at the bottom of these partitions allowing fish to swim into the compartments. The compartments contained food rewards (frozen brine shrimp) placed in glass petri dishes, as well as artificial shelters made from grey 2-mm PVC tubes to reduce the stress associated with exposure to new and open environments. Thus, fish received a double reward (food and shelter) when entering the compartment. For the duration of the experiment, fish were fed exclusively within the maze and were otherwise food deprived, receiving food only as rewards obtained during maze trials.

#### Pretraining

To acclimate fish to the maze, subjects received one 30-min pretraining session per day for three consecutive days prior to the training phase. Fish were netted in their home tanks and gently transferred into the start box using a small glass container. The start box was closed with a PVC screen and an additional screen was placed on top to minimize disturbance. After a 2-min habituation period, the screen and cover were removed, allowing the fish to enter the maze. During pretraining no landmarks were present and both compartments were accessible and contained large amounts of brine shrimp. Individuals that did not voluntarily enter either reward compartment within 20 min during the second and third pretraining sessions were gently guided into a compartment by slowly moving a PVC screen through the maze arms to ensure that they encountered the reward.

#### Training

Training trials were conducted between 0700 and 1700 h in June 2025, with the order of the fish randomized each day. The entire apparatus was surrounded by white curtains to avoid disturbance from the observer and to minimize the availability of global extra-maze cues. Room lighting was used instead of overhead LED lights to minimize reflections from the water surface and improve video quality during recordings. Each fish completed one session of five trials per day. For each session, the initial start box location was pseudorandomly selected with the constraint that no start box was used more than twice in a row. Fish were again given a 2-min habituation period before the start of each trial.

**Fig. 2:**
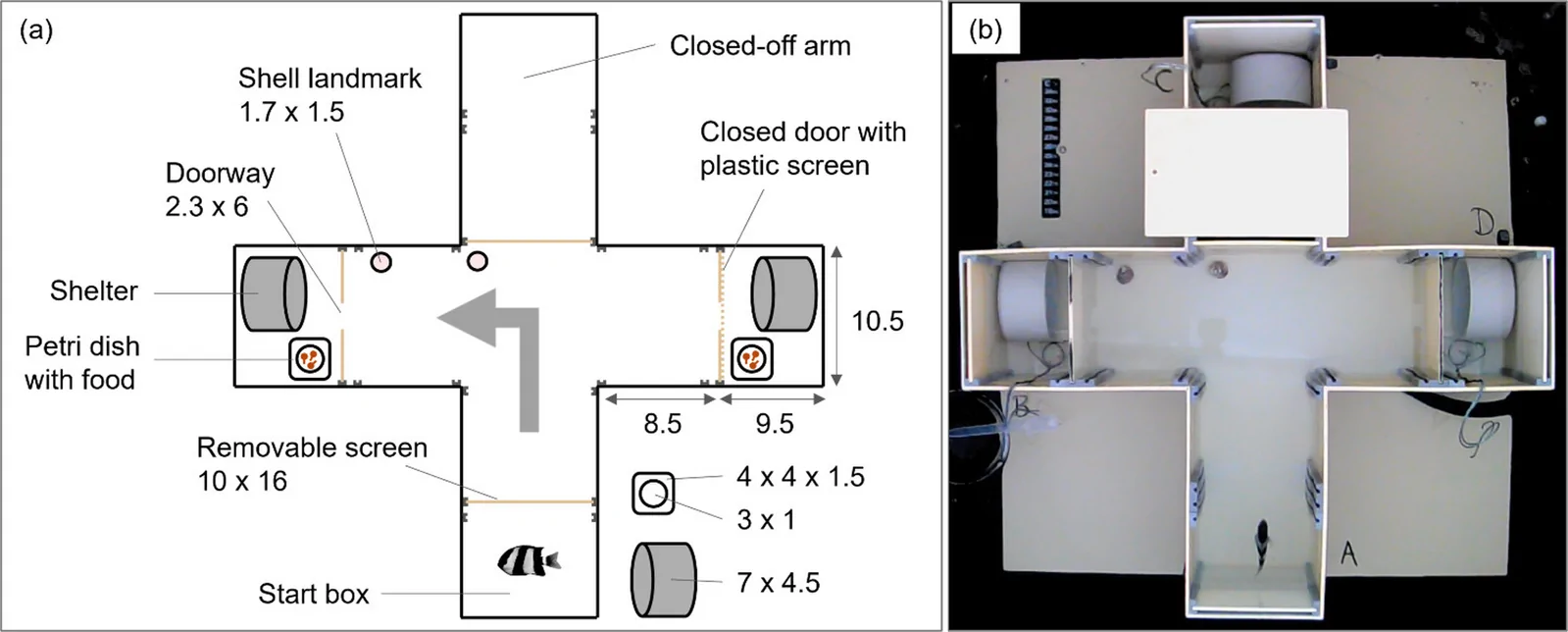
Maze apparatus used in the spatial learning task. (**a**) Schematic drawing (all measurements in cm). Bold arrow indicates correct turn direction (here, left). (**b**) Overhead webcam screenshot of a trial

Half of the fish in each treatment and social rank category were trained to turn right, and half to turn left, to control for any directional bias. Two small shell landmarks were placed inside the maze: one adjacent to the entrance of the reward compartment at the end of the rewarded arm, and the other further along the rewarded arm, where it was visible from the start box (Fig. 2). Subjects could solve the maze using either egocentric (turn) or allocentric (landmark) cues. Both compartments contained the same food (2–3 frozen brine shrimp per trial) and shelter reward, but the unrewarded compartment was rendered inaccessible by attaching a transparent plastic barrier to the back of the doorway. To control for potential olfactory cues, the barrier contained several small perforations, and a similar plastic panel (with the doorway opening cut out) was also attached to the back of the rewarded compartment door. To encourage feeding motivation, a few drops of the water used to defrost food were added to the maze runway. A head bump against the piece of plastic was considered an attempt to enter the unrewarded side. Conversely, if a fish poked its head (including both eyes) through the other, open door, it was considered to have chosen the correct compartment. Trials were scored as successful if the subject chose the correct compartment first, or as failed if no attempt was made or the subject made any incorrect attempts before entering the reward compartment.

Each trial was terminated after the fish had entered the correct compartment, or after 5 min. Fish were left undisturbed for 2 min after entering through the correct doorway. If the subject left the reward compartment during this period, we gently guided it back. A PVC screen was then lowered in front of the doorway and the maze manipulated so that the previously rewarded compartment became the start box for the next trial. The petri dish and shelter were remotely removed using a wire handle and a short fishing line, respectively, to avoid placing hands in the maze and disturbing the fish. In this way, fish rotated around the arms of the maze: anticlockwise (for right-turners) and clockwise (for left-turners). This allowed us to train individuals in blocks of five trials, with minimal handling between trials. It also ensured that fish could not solve the task using fixed extra-maze cues. All fish underwent at least 10 training trials. The learning criterion was established as 8 successful trials out of 10, which corresponds to performance above chance level (binomial test, *p* = 0.044).

#### Probe trials

Upon reaching the learning criterion, subjects were given probe trials to determine their spatial strategy (i.e., turn- or landmark-based). During probe trials, both compartments were accessible and rewarded to avoid punishing the fish for selecting one or other of the available cues. In each probe trial, the landmarks were repositioned to the opposite side from where they had been during training, generating an egocentric/allocentric cue conflict. The probe trials were scored as either turn or landmark choice depending on which compartment the fish chose to enter first. The first probe trial was conducted during the same testing session in which a fish reached the learning criterion, provided that fewer than five trials had been completed that day. If a fish reached criterion on its fifth trial of the day, the first probe trial was conducted during the subsequent testing session on the following day. This ensured that the number of trials experienced by the fish on a given testing day remained consistent, such that the cue contingencies were the primary experimental change. All fish received three probe trials interspersed by additional post-criterion training trials, with a maximum five trials per day. A minimum of 4 successful trials out of 5 had to be achieved between any two probe trials. One subject (T7ReLB; HC, subordinate) did not reach this criterion within 25 post-criterion trials after the second probe trial and was therefore excluded from the probe-trial analysis.

#### Video analysis

All sessions were recorded using a bon.elk HD webcam (SKU: ELK-63022-R; 1080p; 30 fps) mounted 100 cm above the maze and connected to a laptop (AMD Ryzen 5 5500U; 32 GB RAM; Windows Camera app version 2025.2505.2.0). Videos were analysed using BORIS behavioural-analysis software (Friard and Gamba 2016). The following latency measures (in seconds) were extracted: latency to leave the start box, latency to first incorrect and/or correct attempt (during training trials), and latency to first decision (during probe trials).

### Data analysis

All statistical analyses were performed using R Statistical Software (v.4.4.2) in RStudio (v.2025.05.0 + 496). The R script used for the analyses and the raw data are provided in the Supplementary Materials. We used generalized linear mixed models (GLMMs) in the “glmmTMB” package (Brooks et al. 2017) to analyse the number of trials to reach criterion, latency to first correct attempt during training, as well as cue choice (turn vs. landmark) and latency to first decision during probe trials. Random intercepts for fish ID were included to account for repeated measures across trials where applicable. Due to collinearity between environmental treatment and tank, tank number was not included as a random effect. Automated model selection was performed using the *dredge()* function from the “MuMIn” package (Bartoń 2025). Models were ranked based on Akaike’s Information Criterion corrected for small sample size (AICc) and we selected the simplest model with the lowest AICc (see Table 1). Type II Wald chi-square tests were conducted to test whether fixed effects significantly affected the selected models (see Table 2). Model assumptions were verified using the “DHARMa” package (Hartig 2024); no substantial deviations were detected.

We fitted GLMMs with a negative binomial error distribution to analyse trials to criterion in the training phase. Fixed effects included treatment (HC: LC), rank (dominant: intermediate: subordinate), and direction the fish was trained to turn (L: R). Latencies to the first correct attempt were corrected by subtracting the latency to leave the start box area from the raw values. If no correct attempt was made during the trial, the maximum possible latency of 300 s was assigned (*n* = 13 of 437 trials; the main results were robust to exclusion of these trials). Latencies were analysed using GLMMs with a gamma error distribution and a log link function. Fixed effects included environmental treatment, social rank, and direction the fish was trained to turn, as well as two-way interactions between trial and each fixed effect. To improve residual distributions, we used the log of trial number.

Cue choice in the probe trials was coded as a binary variable (turn = 1, landmark = 0). We fitted GLMMs with a binomial distribution and logit link. Fixed effects included treatment, rank, and training turn direction, as well as two-way interactions between probe trial number (1–3) and each fixed effect. Latencies to first decision during probe trials were again corrected by subtracting the latency to leave the start box area and then analysed using GLMMs with a Gamma error distribution and a log link function. Latencies did not differ significantly across the three probe trials (Friedman test: χ²(2) = 0.11, *p* = 0.949); therefore, probe trial number was not included as a fixed effect in model selection. Fixed effects included treatment, rank, training turn direction, and cue choice (landmark: turn) as well as two-way interactions between choice and treatment, and choice and direction. Social rank was not included in interactions to limit model complexity given the small sample size. To further examine environmental treatment effects on decision-making, we conducted non-parametric tests comparing the latency to first decision between treatment groups in the last training trial and first probe trial, separately.

## Results

**Fig. 3:**
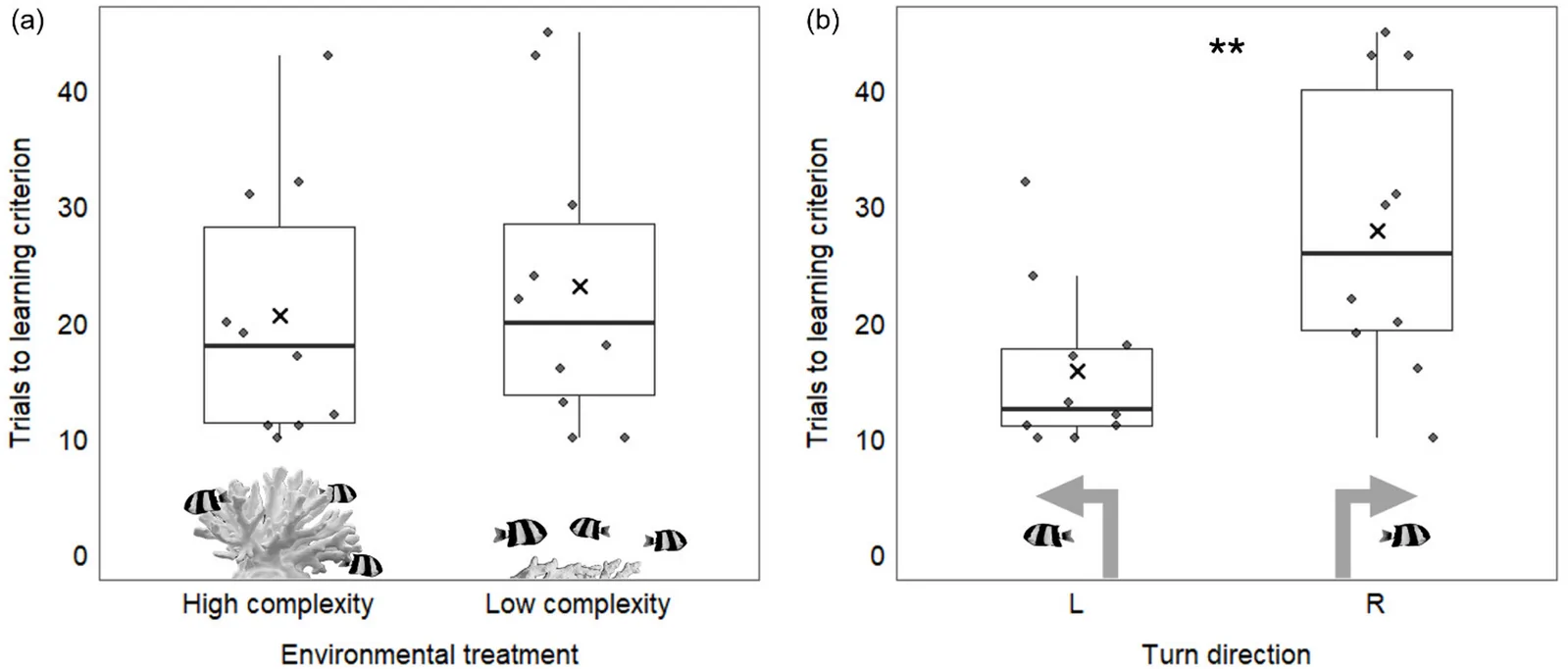
Number of trials to reach the learning criterion, grouped by (**a**) environmental treatment and (**b**) turn direction (L, left; R, right). Boxplots show medians and interquartile ranges, crosses indicate group means. Points represent individual data. Asterisks indicate significant effects

The first set of analyses examined general learning speed, measured as the number of trials individuals needed to reach the learning criterion. The best-supported model included only the direction fish were trained to turn as a fixed effect (Table 1, model A), indicating no statistically significant correlation between learning speed and neither environmental complexity (Fig. 3a) nor social rank. There was a significant effect of turn direction on the number of trials required to reach the learning criterion (χ²(1) = 8.91, *p* = 0.003; Table 2, model A1), with right-turning fish requiring more trials than left-turners (Fig. 3b). This may indicate a left-turn bias in our subjects.

**Fig. 4:**
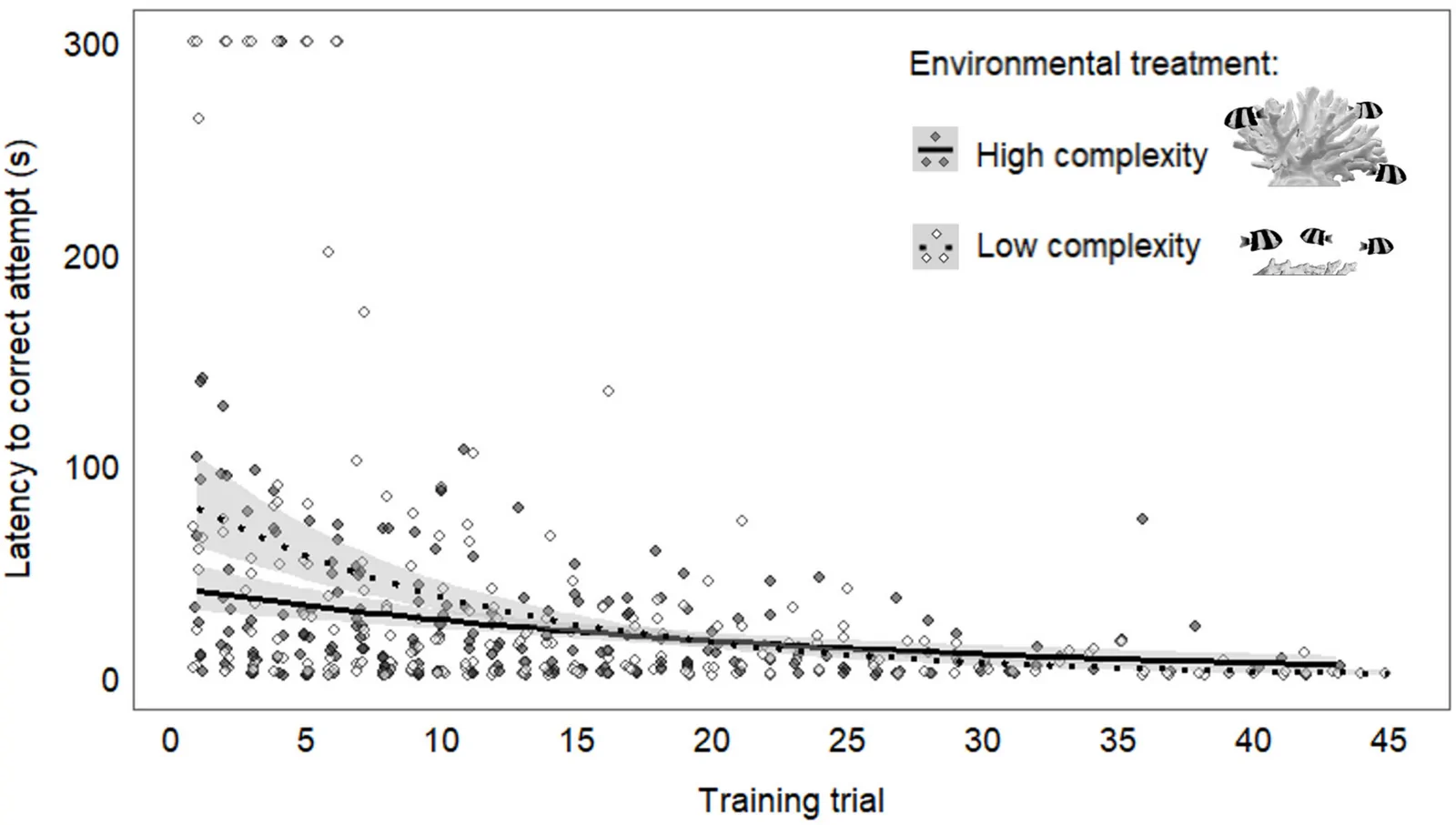
Latency to first correct attempt across training trials for high-complexity (grey points, solid line) and low-complexity (white points, dotted line) fish. Points represent individual data. Lines show model-predicted fitted values (gamma distribution, log link) plotted across raw trial number for ease of interpretation, with 95% confidence intervals

Although environmental treatment did not affect trials to criterion, analysis of latency data revealed that treatment influenced how quickly fish entered the reward compartment across trials. The best-supported model analysing latency to first correct attempt included environmental treatment and its interaction with trial number, as well as a random intercept for fish ID (Table 1, model B). The interaction between trial and treatment significantly affected the latency to first correct attempt (χ²(2) = 107.36, *p* < 0.001; Table 2, model B1), indicating that latencies decreased across trials in both treatment groups, but at different rates. LC fish initially took longer to enter into the reward compartment than HC fish, but this difference diminished over successive trials (Fig. 4).

**Fig. 5:**
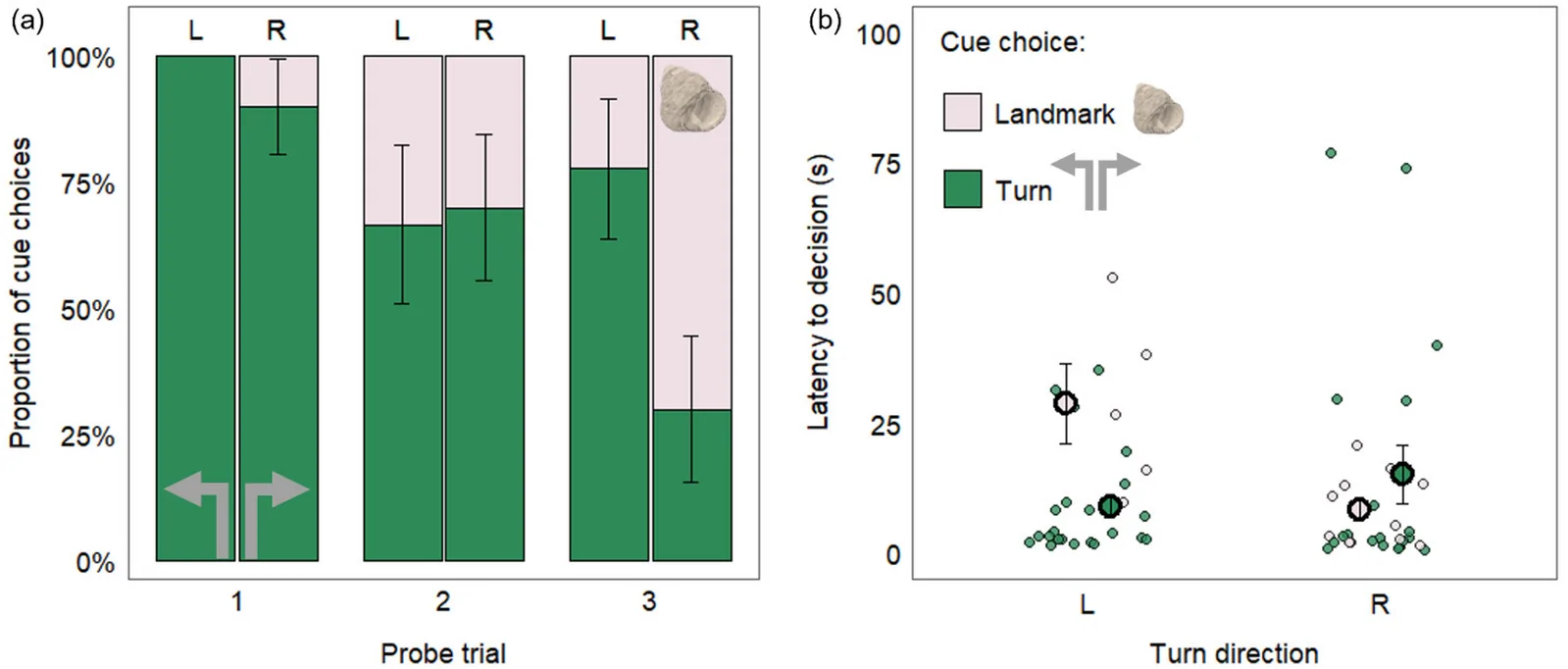
Cue choices and decision latencies during probe trials. Colours correspond to landmark (beige) or turn (green) choices. Error bars indicate standard errors. (a) Proportion of binary choices across the three probe trials, shown separately for fish trained to turn left (L) and right (R). Proportions are calculated from individual-level binary responses (one observation per fish per trial); error bars indicate standard errors from a binomial model and reflect sampling uncertainty. (b) Latency to first decision by cue choice, shown separately for left- and right-trained fish. Small points represent individual data, large points represent group means

To assess fish’s spatial learning strategies, we compared cue choices in the probe trials. Generally, fish relied more on turn cues than landmark cues, with turn cues chosen in 71.8% of trials (*n* = 57). However, this trend varied across probe trials and depended on the direction fish were trained to turn. The best-supported model included turn direction and its interaction with probe trial number, as well as a random intercept for fish ID (Table 1, model C). The interaction between turn direction and trial significantly affected cue choices (χ²(2) = 6.43, *p* = 0.04; Table 2, model C1). Specifically, fish that had been trained to turn right increasingly responded to landmark cues across the three probe trials, whereas left-turners maintained a consistently high probability of using turn cues throughout (Fig. 5a).

Decision latency in probe trials also varied with cue choice and turn direction. The best supported model for probe trial decision latency included cue choice, turn direction, environmental treatment as well as an interaction between cue choice and direction (Table 1, model D) as fixed effects and a random intercept for fish ID. Cue choice significantly affected decision latency (χ²(1) = 5.51, *p* = 0.019; Table 2, model D1) and this effect depended on the direction fish had been trained to turn (χ²(1) = 8.09, *p* = 0.005). Overall, fish made faster decisions when relying on turn cues as opposed to landmark cues. For fish trained to turn left, turn-based decisions were faster than landmark-based decisions, whereas fish trained to turn right were faster when using landmark cues (i.e., when they were turning left during the probe trials; Fig. 5b).

**Fig. 6:**
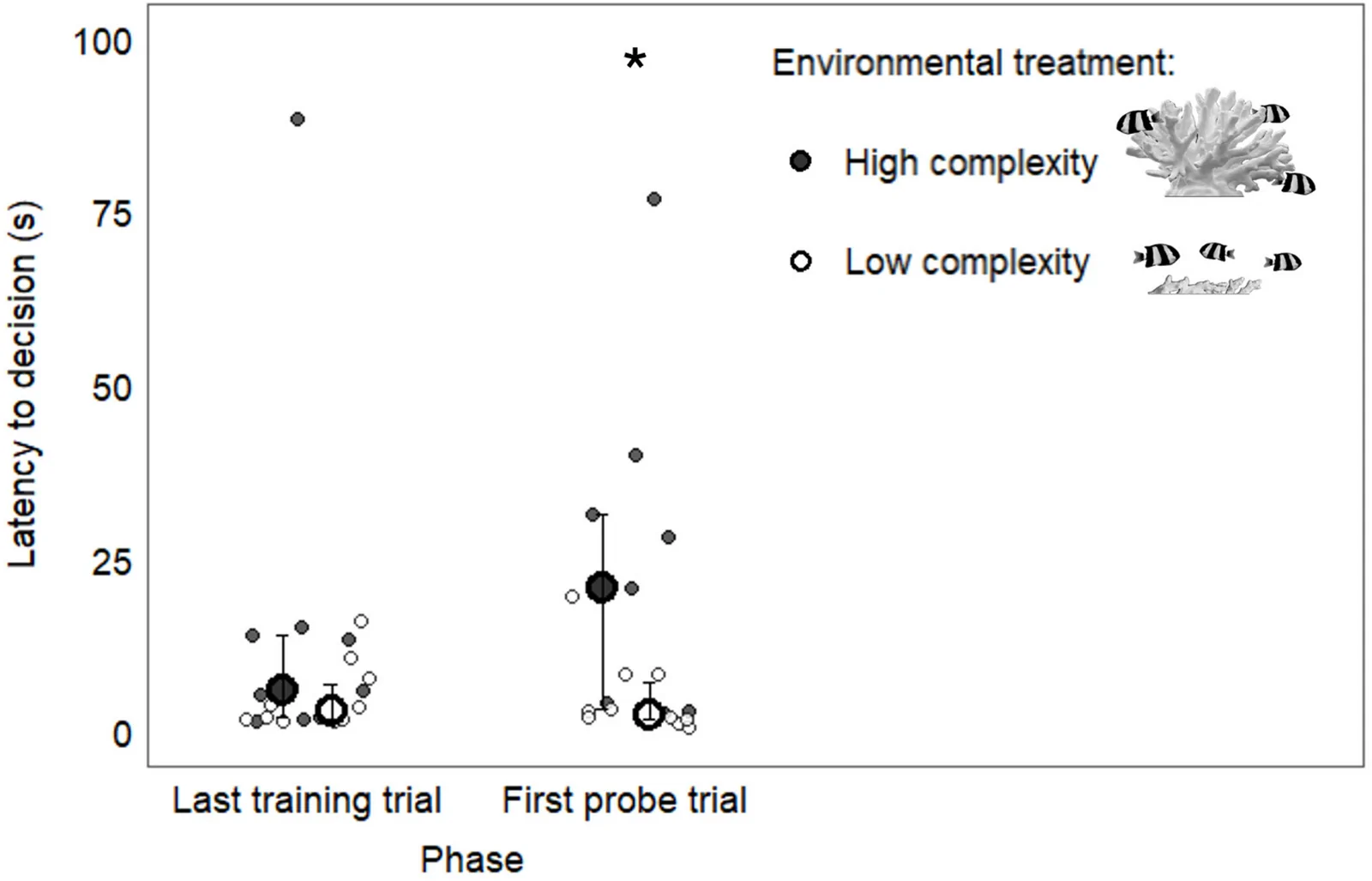
Latency to first decision in each individual’s final training trial and first probe trial, shown separately for high- and low-complexity fish. Small points represent individual data, large points represent group medians. Error bars indicate interquartile ranges. Asterisks indicate significant effects

In the same model, environmental treatment also had a significant effect on decision latency, with fish from the LC treatment making faster decisions during probe trials than fish from the HC treatment (χ²(1) = 15.92, *p* < 0.001). Given that fish from the HC treatment initially located the reward more quickly during training, with this difference diminishing over successive trials (see Fig. 4), we examined whether the observed treatment effect on decision latency in probe trials was related to the change in task contingencies between the final training trial and the first probe trial. We therefore compared decision latency of the two environmental treatment groups during these two trials. During the final training trial, there was no significant difference in decision latency between treatment groups (Mann-Whitney U test: W = 60, *p* = 0.243), indicating similar decision speeds by HC and LC fish at the end of training. In contrast, during the first probe trial, fish from the HC treatment showed significantly longer decision latencies than LC fish (Mann-Whitney U test: W = 75, *p* = 0.013*), indicating a difference in behaviour between treatment groups in response to the change in task contingencies (Fig. 6).

**Table 1:** Model selection results for generalized linear mixed models analysing (A) trials to learning criterion (training), (B) latency to correct attempt (training), (C) cue choice (probe trials), and (D) latency to first decision (probe trials). For each response variable, the five best-supported candidate models derived from the corresponding global model are shown. Models were ranked using AICc; ΔAICc values are relative to the best-supported model within each panel. Akaike weights indicate relative model support. Fish ID was included as a random intercept where indicated. Models selected for interpretation are shown in bold. To analyse the latency to correct attempt (B), we used the log of trial number to improve residual distributions

Model	Fixed effects	df	logLik	AICc	ΔAICc	Akaike weight
(A) Global model: Trials to criterion ~ Treatment + Social rank + Direction, family = negative binomial						
**A1**	**Direction**	**3**	**-70.953**	**149.4**	**0.00**	**0.479**
A2	Direction + Social rank	5	-68.067	150.4	1.01	0.289
A3	Direction + Treatment	4	-70.863	152.4	2.99	0.108
A4	Intercept (null model)	2	-74.662	154.0	4.62	0.047
A5	Direction + Social rank + Treatment	6	-67.946	154.4	4.95	0.040

(B) Global model: Latency to correct attempt ~ Treatment + Social rank + Direction + logTrial: Treatment + logTrial: Social rank + logTrial: Direction + (1 | ID), family = gamma (log)						
**B1**	**Treatment + logTrial: Treatment**	**6**	**-1791.098**	**3594.4**	**0.00**	**0.240**
B2	Treatment + Social rank + logTrial: Treatment	8	-1789.175	3594.7	0.30	0.207
B3	Treatment + Direction + logTrial: Treatment	7	-1790.829	3595.9	1.53	0.112
B4	Treatment + Social rank + Direction + logTrial: Treatment	9	-1788.846	3596.1	1.72	0.101
B5	Treatment + Social rank + logTrial: Treatment + logTrial: Social rank	10	-1788.293	3597.1	2.71	0.062

(C) Global model: Cue choice ~ Treatment + Social rank + Direction + Trial: Treatment + Trial: Social rank + Trial: Direction + (1 | ID), family = binomial (logit)						
**C1**	**Direction + Trial: Direction**	**5**	**-27.305**	**65.8**	**0.00**	**0.305**
C2	Treatment + Trial: Treatment	5	-27.864	66.9	1.12	0.174
C3	Direction + Treatment + Trial: Treatment	6	-26.786	67.3	1.47	0.146
C4	Direction + Treatment + Trial: Direction	6	-26.996	67.7	1.89	0.119
C5	Direction + Treatment + Trial: Direction + Trial: Treatment	7	-26.201	68.7	2.90	0.071

(D) Global model: Latency to first decision ~ Treatment + Social rank + Direction + Cue choice + Choice: Treatment + Choice: Direction + (1 | ID), family = gamma (log)						
**D1**	**Treatment + Direction + Cue choice + Choice: Direction**	**7**	**-177.148**	**370.6**	**0.00**	**0.347**
D2	Treatment + Direction + Cue choice + Choice: Direction + Choice: Treatment	8	-176.192	371.4	0.80	0.233
D3	Treatment + Direction + Cue choice + Social rank + Choice: Direction	9	-175.217	372.3	1.68	0.150
D4	Treatment + Direction + Cue choice + Social rank + Choice: Direction, Choice: Treatment	10	-174.256	373.3	2.71	0.089
D5	Treatment + Cue choice + Choice: Treatment	6	-180.668	375.0	4.43	0.038

AICc, Akaike Information Criterion corrected for small sample size; df, degrees of freedom; HC, high-complexity treatment; LC, low-complexity treatment; logLik, log-likelihood

**Table 2:** Parameter estimates and analysis of deviance table using type II Wald chi-square tests for selected generalized linear mixed models. Estimates are shown on the link scale. Reference levels were: Treatment = HC, Direction = L, Social rank = subordinate, and Cue choice = landmark

Model	Predictor (level)	Estimate	SE	z	*p*	Wald χ²	df	*p*
**A1 Trials to learning criterion (training)** *Trials to criterion ~ Direction*,* (negative binomial)*	(Intercept)	2.7600	0.1397	19.756	< 0.001			
	Direction (R)	0.5686	0.1905	2.985	0.003	8.9098	1	0.003 **
**B1 Latency to correct attempt (training)** *Latency to correct attempt ~ Treatment + logTrial: Treatment + (1 | ID)*,* (gamma (log))*	(Intercept)	4.24870	0.30121	14.106	< 0.001			
	Treatment (LC)	0.56839	0.43315	1.312	0.189	0.0943	1	0.7588
	Treatment (HC): logTrial	-0.51799	0.09005	-5.752	< 0.001	107.3595	2	< 0.001 ***
	Treatment (LC): logTrial	-0.81873	0.09498	-8.620	< 0.001			
**C1 Cue choice (probe trials)** *Response ~ Direction + Trial: Direction + * *(1 | ID)*,* (binomial (logit))*	(Intercept)	3.6565	1.8868	1.938	0.0526			
	Direction (R)	0.9115	2.4052	0.379	0.7047	0.8540	1	0.355
	Direction (L): Trial	-0.8846	0.7270	-1.217	0.2237	6.4319	2	0.040 *
	Direction (R): Trial	-1.8417	0.7878	-2.338	0.0194			
**D1 Latency to first decision (probe trials)** *Latency to first decision ~ Treatment + Direction + Cue choice + Choice: Direction + * *(1 | ID)*,* (gamma (log))*	(Intercept)	4.1654	0.4506	9.244	< 0.001			
	Treatment (LC)	-1.3662	0.3424	-3.990	< 0.001	15.9181	1	< 0.001 ***
	Direction (R)	-1.5374	0.5054	-3.042	0.002	1.9875	1	0.159
	Cue choice (turn)	-1.4815	0.4022	-3.684	< 0.001	5.5124	1	0.019 *
	Cue choice (turn): Direction (R)	1.4162	0.4978	2.845	0.004	8.0922	1	0.005 **

Significance codes: * *p* < 0.05, ** *p* < 0.01, *** *p* < 0.001. Abbreviations: df, degrees of freedom; HC, high-complexity treatment; L, left; LC, low-complexity treatment; R, right; SE, standard error

## Discussion

The aim of this study was to test whether environmental complexity influenced spatial learning and navigation strategies in humbug damselfish. We predicted that fish reared under structurally complex environmental treatments (HC) would learn faster and rely more strongly on allocentric (landmark) cues than those from simpler environments (LC). Although habitat complexity did not affect the number of trials to criterion, it influenced decision latencies, including when cues conflicted in probe trials, suggesting effects on information processing and cue use rather than learning speed per se. Instead, turn direction was the main driver of both learning speed and navigational strategy use. Fish that were trained to turn left solved the task in fewer trials than right-turning fish and consistently relied on egocentric (turn) strategies, indicating a general left-turn bias. In contrast, fish trained to turn right increasingly relied on landmark cues during probe trials. While fish generally made faster decisions when using turn cues, this was reversed for those trained to turn right who used landmarks.

### Effects of turn direction on learning speed

Contrary to our expectation that habitat complexity would influence learning, the strongest determinant of learning speed in this study was instead turn direction. Fish trained to turn left reached criterion faster than right-turning fish, indicating an underlying, population-level lateralisation bias. Although we did not explicitly quantify cerebral lateralisation, turn biases are commonly used as behavioural proxies for lateralisation in fishes and other vertebrates (Bisazza and Brown 2011). Additionally, in an unpublished inhibitory-control assay conducted by our research group (Klinke and Brown, in review) in which the same individuals repeatedly had to pass a barrier on either the left or right side across nine trials, of those that had a significant side bias (40% of the population), most fish showed a preference to the left side, with only a single individual exhibiting a significant right-side bias. This suggests that turn biases were consistent across tasks and not solely induced by the maze procedure.

Population-level lateralisation is most commonly observed in highly social species (Bisazza et al. 2000; Vallortigara and Rogers 2005), suggesting that alignment in turn biases could be a consequence of coordinating complex social behaviour (Rogers 1989). In our study, the left-turn bias was consistent across environmental treatment groups, and because humbug damselfish are highly social, this pattern is consistent with the idea that social factors can shape population-level lateralisation. However, although all individuals were collected from the same reef, we lack information on their genetic relatedness. Given that lateralisation can have genetic components (Bisazza and Lucon-Xiccato 2025), we cannot infer whether the observed bias reflects genetic, environmental, or social processes.

In conditions under pressure or increased stress, strongly lateralised individuals often outperform weakly lateralised individuals (Rogers et al. 2004; Dadda and Bisazza 2006; Dadda et al. 2010), possibly because lateralisation enhances decision-making efficiency by reducing neural interference and allowing simultaneous processing of sensory and motor information (Bisazza and Brown 2011). In this study, the manipulation of the maze during training sessions may have increased stress or perceived risk in the subjects. The poorer performance of right-turning fish likely reflects the cost of overriding a habitual turn bias in a stressful setting. Strong lateralisation can negatively affect performance in maze tasks (Pellitteri-Rosa et al. 2025). When Brown and Braithwaite (2005) tested poecilids from low- and high-predation areas in a spatial foraging task, high-predation fish performed less efficiently due to a consistent turn bias. Thus, spatial learning might be impaired by lateralisation if animals are required to act against their preferred turn direction, and in therefore, performance differences may be due to these constraints rather than cognitive ability.

### Does lateralisation impact the efficiency of navigation strategies?

Turn direction also affected which spatial strategies fish used. Overall, fish more frequently relied on egocentric navigation, but strategy use varied with turn direction across probe trials. Fish trained to turn right progressively shifted from turn-based towards landmark-based choices. Using allocentric cues allowed this group to turn left at the decision point. This pattern suggests that strategy use was not fixed after the training phase, but changed as fish learned about the altered task contingencies during the probe trials.

Several studies have reported that strategy use can be flexible. Rats do not exclusively rely on either place (landmark) or response (turn) learning when navigating mazes; they typically use all relevant cues and use whichever is most useful in a given context (Restle 1957). Similarly, fishes and other vertebrates can use egocentric and allocentric cues independently or integrate both to optimize navigation (Huntingford and Wright 1989; Rodríguez et al. 2002, 2021; Lunt et al. 2022).

While all fish initially relied on a habitual egocentric rule after training, fish trained to turn right likely shifted to allocentric cues over the three probe trials because they learned that when the landmark had been moved, this information could be used to follow their preferred turn direction. We found some support for this hypothesis when we looked at the speed at which decisions were made. During probe trials, turn-based decisions were generally faster than landmark-based decisions. If egocentric mechanisms form a subsystem of allocentric navigation rather than a fully independent pathway (Wang et al. 2020; Sibeaux et al. 2025), they could be cognitively simpler to implement than allocentric strategies, which require integrating external cues. However, for right-turning fish, constraints imposed by lateralisation may have outweighed these benefits. This group made decisions faster when relying on landmark cues, indicating that the switch to allocentric strategies reduced the cost of overriding their intrinsic turn bias.

These results suggest that turn biases can influence which spatial strategies are used and how efficiently they are applied. To the best of our knowledge, this is the first evidence that cerebral lateralisation could potentially influence the relative efficiency of egocentric and allocentric navigation strategies. Rather than relying on fixed navigation strategies, fish appear able to flexibly update how different cue types are weighted and to switch between strategies in ways that maximise efficiency.

### Effects of habitat complexity on learning and task engagement

Contrary to our expectations, environmental complexity did not influence learning speed. This contrasts with several studies reporting enhanced cognitive performance in fishes from structurally complex or enriched environments (e.g., White and Brown 2015; Fong et al. 2019; Iffert and Stein 2024), although the direction of the effect has varied among studies (Irwin et al. 2024). Differences among species, tasks, and measures of environmental complexity may contribute to these different outcomes. One possible explanation for the limited treatment effects is that the contrast between habitats was insufficiently large. However, our treatments were designed to reflect realistic variation in coral habitat structure experienced by *D. aruanus* rather than an extreme comparison between complex habitat and a completely barren environment. Additionally, environmental and social factors were not fully independent in our experimental design. While brain development can be shaped by environmental complexity, it is also influenced by social factors (Shumway 2008). In our study, the increased social density in LC tanks could have driven neural development, potentially confounding treatment effects. A further limitation is the relatively small sample size, particularly given the multiple sources of variation in the experiment. Individual turn direction, social rank, and other behavioural differences may contribute to variation in spatial performance, reducing our ability to detect comparatively small effects of habitat complexity. Thus, the absence of a treatment effect does not necessarily indicate that habitat complexity has no influence on spatial cognition in this species.

Performance in cognitive tasks can reflect more than just learning ability and involves at least two distinct stages: encountering the task contingency and subsequently learning to follow the association (Shettleworth 2010). Although habitat complexity did not affect learning speed per se, treatment groups behaved differently in early stages of the learning process, with HC fish initially entering the reward compartment faster than LC fish. This likely reflects differences in personality or motivation to engage with the task. Individual differences in behaviour, or personality, can affect cognitive performance (Coppens et al. 2010; Carere and Locurto 2011; Sih and Del Giudice 2012). In the present study, the reward consisted of both food and shelter, and motivation to access shelter could have differed between treatment groups. Fish from HC treatments were accustomed to using shelter extensively in the form of their home corals, whereas LC fish were used to limited refuge availability. As a result, HC fish may have been more motivated to locate the shelter during the first trials when the apparatus and training procedure were still unfamiliar. By the end of training, all subjects reached similar latencies, suggesting that fish valued this reward equally once the task was learned and they had habituated to the test arena.

### Differences in spatial information use across low- and high-complexity fish

We hypothesized that fish from HC treatments would rely more strongly on allocentric strategies. While we found no treatment effect on cue choice per se, HC fish made slower decisions during probe trials despite similar decision latencies between treatments at the end of training. The only difference between the final training trial and the first probe trial was that landmarks were moved to the opposite arm, creating a conflict between the learned turn direction and landmark cues. The longer decision times of HC fish may therefore indicate hesitancy caused by conflicting cues, suggesting they paid greater attention to landmark information than fish from the LC treatment. An alternative explanation is that HC fish simply responded more cautiously to the changed conditions of the probe trials. However, this interpretation seems less likely given that HC fish were initially faster to locate the reward compartment and did not show longer decision latencies during training. Thus, their slower decisions specifically in the first probe trial may instead reflect greater processing of, or attention to, the conflicting spatial cues. Importantly, a choice consistent with one strategy does not imply that other learning has not occurred (Shettleworth 2010). HC fish may have considered both information sources in parallel while still predominantly making choices based on egocentric cues, whereas the faster decisions of LC fish suggest they relied on a single turn-based rule. This could reduce the energetic and neural costs associated with integrating multiple cue types (De Meester et al. 2025). Given the high metabolic cost of brain tissue, selectively investing only in the cognitive mechanisms required for a given habitat could be an adaptive trade-off (Mery and Kawecki 2003; Brown and Braithwaite 2005).

In gobies, species from low-complexity sand flats rely predominantly on turn responses, whereas rock-pool species from structurally complex habitats use both egocentric and allocentric information (White and Brown 2015). Three-spined sticklebacks from still ponds and flowing rivers also differ in their use of landmarks: pond fish use both turn-direction and landmark cues, whereas river fish rely mostly on turn responses (Odling-Smee and Braithwaite 2003a). From an ecological perspective, reliance on egocentric strategies alone may be sufficient in environments with low landmark availability, where integrating multiple cues offers little additional benefit and may also be more cognitively demanding (Wang et al. 2020; Sibeaux et al. 2025). Although coral reefs are landmark-rich and spatially heterogeneous ecosystems, reliance on landmarks alone may be insufficient under conditions of reduced visibility or disturbance (e.g., coral growth or erosion, storm damage, algal blooms; Lunt et al. 2022). Damselfish have been shown to combine self-generated and external cues to navigate efficiently (Murley et al. 2024). High-complexity environments could favour the use of multiple spatial information sources because more information is available and required to navigate efficiently.

## Conclusion

Variation in maze performance in humbug damselfish was better explained by turn biases and how individuals use different types of spatial information than by differences in environmental complexity. Because such factors do not necessarily reflect differences in learning capacity, our findings highlight the importance of considering individual-level traits and fine-scale behavioural measures when interpreting cognitive performance.

## Supplementary Information

Below is the link to the electronic supplementary material.

Information on individual fish, including treatment, bodysize (in cm, measured in October 2024 and again in May 2025), and social rank (R1, dominant; R2, intermediate; R3, subordinate). Individual IDs encode tank number (T…) and the colour (either Gr, green or Re, red) and position (either L, left or R, right and F, front or B, back) of the VIE tags of individual fish in each tank.

Raw data used for the analyses in this study.

R script used to perform the analyses in this study.

## Data Availability

Raw data obtained from video analysis were deposited into the Figshare database and are available at the following URL: https://doi.org/10.6084/m9.figshare.31418465.
